# Cold resistance of mammalian hibernators ∼ a matter of ferroptosis?

**DOI:** 10.3389/fphys.2024.1377986

**Published:** 2024-04-25

**Authors:** Masamitsu Sone, Yoshifumi Yamaguchi

**Affiliations:** ^1^ Hibernation Metabolism, Physiology and Development Group, Institute of Low Temperature Science, Hokkaido University, Sapporo, Japan; ^2^ Graduate School of Environmental Science, Hokkaido University, Sapporo, Japan

**Keywords:** cold resistance, hibernation, torpor, homeotherm, cell death, ferroptosis

## Abstract

Most mammals adapt thermal physiology around 37°C and large deviations from their range, as observed in severe hypothermia and hyperthermia, resulting in organ dysfunction and individual death. A prominent exception is mammalian hibernation. Mammalian hibernators resist the long-term duration of severe low body temperature that is lethal to non-hibernators, including humans and mice. This cold resistance is supported, at least in part, by intrinsic cellular properties, since primary or immortalized cells from several hibernator species can survive longer than those from non-hibernators when cultured at cold temperatures. Recent studies have suggested that cold-induced cell death fulfills the hallmarks of ferroptosis, a type of necrotic cell death that accompanies extensive lipid peroxidation by iron-ion-mediated reactions. In this review, we summarize the current knowledge of cold resistance of mammalian hibernators at the cellular and molecular levels to organ and systemic levels and discuss key pathways that confer cold resistance in mammals.

## Life in the cold

In a cold environment, homeotherms including birds and mammals can maintain their core body temperature (Tb) higher than ambient temperature because of the homeostatic system of body temperature and high thermogenic capacity, thereby being able to operate at night and expand ecological niches into temperate and even polar climates. However, most homeotherms are vulnerable to drastic changes in the Tb. The core Tb of homeotherms is maintained within a value of 1°C, and in many cases, around 37°C. If an insufficient energy source for maintaining Tb homeostasis is obtained for the long term, it finally results in lowered Tb and subsequently malfunction of organs and the whole body system, threatening their lives in strict homeotherms. In contrast, some homeotherms can dynamically change their Tb in a highly regulated manner by being liberated from the restriction of keeping Tb constant and are called heterotherms because of this feature (Ruf and Geiser, 2015). Among heterothermic events, torpor and hibernation are the most prominent examples of drastic changes in Tb (Ruf and Geiser, 2015; Giroud et al., 2020). During torpor, animals suppress their basal metabolic rate and exhibit a very low Tb. Torpor that occurs for a short period (less than 24 h) in response to fasting (starvation) or other environmental fluctuations that demand animals to reduce energy consumption in an opportunistic manner is classified as daily torpor. Torpor that lasts over 24 h with a very low Tb (often defined below 10°C or 15°C) is called a deep torpor. When deep torpor occurs repeatedly and seasonally for a long period, it is called hibernation. Torpor and hibernation have been observed across all mammalian clades as adaptive mechanisms to cope with fluctuating environments. Hereafter, we use the words “hibernators” for mammals that are able to hibernate among heterotherm and “non-hibernators” for mammals other than hibernators. How torpor and hibernation were acquired during evolution and what discriminates hibernators from non-hibernators or heterotherms from homeotherms are long-lasting questions and are still a matter of debate (Grigg et al., 2004; Canale et al., 2012; Boyles et al., 2013; Ruf and Geiser, 2015; Giroud et al., 2020). Dissecting the physiological, genetic, and molecular mechanisms of torpor and hibernation would help clarify these questions.

## Cell-intrinsic cold resistance

One of the important factors that enables hibernation and discrimination of hibernators from non-hibernators is cold resistance, the ability to endure extreme and prolonged low Tb experienced during a long hibernation period. It has long been known that cold resistance is not observed in non-hibernators, including strict homeotherms, such as humans, and heterotherms, such as mice, not only at whole body level but also at tissues and cellular levels (Lyman et al., 1982); When exposed to extreme cold temperatures lower than 10°C, primary cultured cells or tumor cells from non-hibernators such as humans, mice, and rats die within a day or several days, but those from hibernators can survive for over 5 days (Hendriks et al., 2017; Ou et al., 2018; Hendriks et al., 2020; Anegawa et al., 2021). Such difference in cold resistance between hibernators and non-hibernators is also observed in neurons and hepatocytes differentiated from induced pluripotent stem cells (iPSC) derived from a hibernator thirteen-lined ground squirrels (13LGS) (*Ictidomys tridecemlineatus*), highlighting the cell-intrinsic properties of cold resistance and cellular homeostasis in hibernators (Ou et al., 2018; Zhang et al., 2022). Interestingly, such cell-intrinsic properties to sustain cellular homeostasis under cold conditions may also be observed in embryonic stem (ES) cells of mice (*Mus musculus*), which do not hibernate but do fasting-induced torpor (FIT), and are therefore classified as heterotherms. Distinct mouse strains exhibit FIT with different torpor depths depending on the strains; the STM2 strain (*Mus musculus molossinus*) exhibits the lowest Tb of 23°C, whereas B6J (*Mus musculus musculus*) and MYS/Mz (*Mus musculus castaneus*) exhibit Tb of 31°C and 34°C, respectively (Suita et al., 2023). Interestingly, ES cells and liver cells from STM2 strains can sustain oxidative phosphorylation (OxPhos) and higher oxygen consumption at lower temperatures (31°C), whereas those from B6J or MYS/Mz inactivate OxPhos and metabolically incline to glycolysis, suggesting that the cell-intrinsic ability of STM2 mice to maintain mitochondrial activity at low temperatures may enable its lowest Tb in FIT. Thus, the lowest temperature limit that animals can withstand at the cellular level may be a genetically defined intrinsic trait in heterotherms, including both hibernators and non-hibernators.

## Dying in cold ∼ a matter of ferroptosis?

Why, in the first place, do most mammalian cells die when exposed to low temperature for long duration, though duration and temperature for triggering cold-induced cell death differs among animals and cell types? A classical scheme of how prolonged low temperature leads to cell death was proposed long ago (Hochachka, 1986; Aloia and Raison, 1989; Boutilier, 2001; Rubinsky, 2003). At low temperatures, the lipid membrane bilayers of cells undergo a phase transition into undesired forms, such as a gel phase, leading to segregation of membrane lipids and proteins, and membrane damage. Because of such chronic membrane damage and leakage, ion gradients across cell membranes cannot be sustained, resulting in an increased net Na^+^ influx and K^+^ efflux. Dysregulation of Na^+^/K^+^ homeostasis by low temperature was proposed to be caused by failure of ATP homeostasis (Boutilier, 2001) and differences in the temperature coefficient between ion-channel-mediated efflux of K^+^ and Na^+^/K^+^ -ATP pump-dependent influx of K^+^ (Hochachka, 1986). As a result, voltage-dependent calcium channels open, and calcium ion concentrations increase within cells. The increased calcium ion concentration further activates calcium ion-dependent phospholipases and proteases and causes remodeling of the plasma membranes, leading to cell death (Hochachka, 1986). Biochemical studies on key metabolic enzymes involved in ion homeostasis, such as Na^+^ -K^+^ -ATPase, identified differences in the kinetics of hibernators such as ground squirrels and non-hibernators such as rats (Lyman et al., 1982). However, such differences do not sufficiently explain the remarkable cell-intrinsic cold resistance of hibernators. Living organisms also adopt a mechanism called “homeoviscous adaptation,” which is the phenomenon of cells adjusting the viscosity of the membrane lipids and thereby compensating membrane fluidity to different temperatures. It includes cold-induced decreases in the length and/or increases in the unsaturation of the fatty acyl chains and shifts in phospholipid composition (Aloia and Raison, 1989). However, little is known about the exact contribution of homeoviscous adaptation to hibernators’ cold resistance. Resistance to various other stresses such as hypoxia/reoxygenation and ischemia/reperfusion are also observed in hibernators (Dave et al., 2012). Thus, there is much room for considering the additional mechanisms of why and how mammalian cells are sensitive to cold temperatures from the current perspective.

**FIGURE 1:**
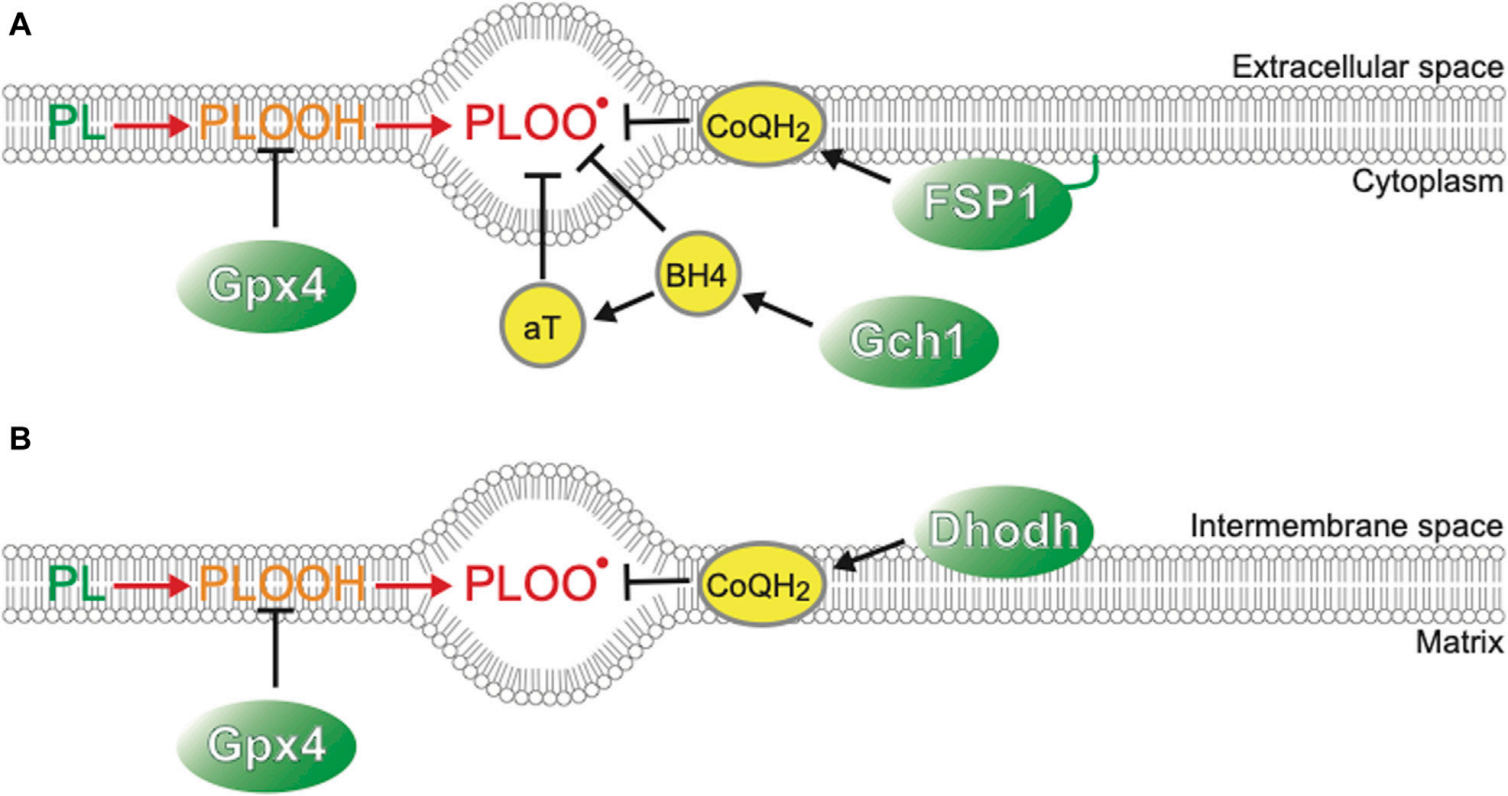
Players suppressing ferroptosis **(A)** Plasma membrane. **(B)** Mitochondrial inner membrane.

Cell death can be classified into many types according to the molecular, cellular, and morphological features during the dying process and functional assessment with genetic and/or pharmaceutical inhibition of each cell death type (Galluzzi et al., 2018). Cold-induced cell death in cancer cell lines and primary hepatocytes can be regarded as ferroptosis, a form of necrotic cell death with hallmarks such as necrotic morphology, iron dependency, and extensive lipid peroxidation (Hattori et al., 2017; Hendriks et al., 2017; Hendriks et al., 2020; Anegawa et al., 2021). Ferroptosis was formally named in 2012 as a novel form of cell death, which was induced in certain types of cancers by the anti-cancer drug erastin, which targets the xCT system to transport cystine into cells and has distinct features from other types of cell death, such as apoptosis or classical necrosis (Seiler et al., 2008; Dixon et al., 2012; Stockwell and Jiang, 2020). Cystine is a source of glutathione synthesis, and inhibition of this system results in inadequate activity of glutathione peroxidase 4 (GPx4), which reduces lipid peroxide to lipid alcohol and thereby acts as a repair system for damaged cellular membranes. GPx4 deficiency gives rise to ferroptosis in an iron-dependent manner (Figure 1). First, hydrogen extraction occurs in phospholipids containing polyunsaturated fatty acyl tails by reactive oxygen species (ROS), such as hydrogen peroxide (H_2_O_2_) and superhydroxyl radical (-OH), or iron-dependent peroxidation enzymes, such as lipoxygenases (Yang et al., 2016; Wenzel et al., 2017) and cytosolic cytochrome P450s (Zou et al., 2020; Yan et al., 2021), leading to further oxidization of lipid radicals by O_2_ into lipid peroxyl radicals. Once lipid peroxyl radicals are formed, they extract hydrogen from nearby unsaturated fatty acids, resulting in the formation of lipid radicals and lipid peroxides. Although lipid peroxides are relatively stable, they can facilitate iron-mediated Fenton reactions or other reactions that generate new lipid radicals. As a result, two molecules of lipid radicals are produced from one molecule per cycle, leading to an exponential increase in the number of lipid radicals. This chain reaction of lipid oxidation propagates to the cell membrane or organelle membrane and finally destroys membrane structures, resulting in necrotic morphology (Yan et al., 2021). As labile Fe^2+^ in cytoplasm mediates harmful Fenton reaction, its concentration is tightly regulated to be as low as <5% of total cellular iron, the rest of which is incorporated into Ferritin, a protein that oxidizes Fe^2+^ to Fe^3+^ irons and store them in its cage-like structure, Fe-S clusters, and heme (Kakhlon and Cabantchik, 2002; Galy et al., 2024). Extracellular iron is imported into cells mainly through an iron-carrier protein Transferrin and its receptor TfR, or alternatively through other routes such as metal cation symporter ZIP14 (Galy et al., 2024). Modulation of these Fe^2+^ regulatory systems leads to the changes of cellular susceptibility to ferroptosis inducers; for example, inhibition of NCOA4, which mediates autophagic degradation of Ferritin and therefore increases availability of labile Fe^2+^, leads to resistance to a ferroptosis inducing drug, erastin (Gao et al., 2016; Hou et al., 2016). The mechanisms of ferroptosis have been extensively studied in the field of cancer biology, because some cancer cells exhibit susceptivity to ferroptosis and revealing its mechanisms may provide novel therapeutic approaches against tumors that are resistant to conventional anti-cancer drugs that target well-known regulated cell death pathways such as apoptosis. Several crucial pathways to prevent/accelerate ferroptosis have been found (Figure 1).

## Possible mechanisms of cold-induced ferroptosis

As mentioned above, clues to the molecular mechanisms of cold-induced ferroptosis have come from human cancer studies. Cold stress (4°C) activates the apoptosis signal-regulating kinase 1 (ASK1)-p38 MAPK signaling cascade, which is activated by oxidative stress and reactive oxygen species (ROS). Inhibition of the ASK1-p38 pathway delays cell death under cold temperatures in multiple human cancer cell lines (Hattori et al., 2017). Cold-induced cell death in human cancer cells is not inhibited by inhibitors of apoptosis, a well-known regulated cell death mediated by the activation of caspases, which are evolutionarily conserved cysteine proteases, but is inhibited by an iron-chelator deferoxamine and ferrostatin-1, potent inhibitors of ferroptosis, leading to the notion that cell death could be regarded as cold-induced ferroptosis. Loss-of-function screening for suppressors of cold-induced ferroptosis in human cancer cell lines identified mitochondrial calcium uptake protein 1 (MICU1), a protein located in the inner mitochondrial membrane that is involved in the transport of Ca^2+^ to the mitochondrial matrix (Nakamura et al., 2021). Human cancer cell lines lacking the MICU1 gene do not exhibit an increase in Ca^2+^ concentration in the mitochondrial matrix under short-term (16 h) cold (4°C) culture and suppress mitochondrial membrane potential (MMP) hyperpolarization just after cold exposure, lipid peroxidation, and subsequent cell death, all of which are triggered by cold. Thus, MICU1 is involved in cold-induced ferroptosis in certain types of cancer cells. The mechanism by which MICU1 deficiency suppresses cold-induced ferroptosis has not been fully elucidated, but one possible scenario proposed is as follows: cold induces calcium influx into the cytosol, leading to an increase in Ca^2+^ concentration in the cytosol and subsequently in the mitochondria via MICU1. This Ca^2+^ influx causes an increase in the activity of electron transport complexes (ETC), leading to mitochondrial membrane (MMP) hyperpolarization, which facilitates ROS generation at multiple steps of, ETC, resulting in lipid peroxidation. A similar scenario was proposed for cultured neurons (Ou et al., 2018). Neurons differentiated from human iPSC exhibit MMP hyperpolarization after cold exposure, then lose MMP and finally die, but depolarization by inhibitors of, ETC complexes, ionophores, or overexpression of mitochondrial uncoupler proteins that cause proton leak suppresses cell death. Thus, it is likely that a cascade of cytosolic calcium increases, and mitochondrial hyperpolarization, subsequent ROS generation, and lipid peroxidation is vital for cold-induced ferroptosis.

On the other hand, studies in cancer cells cultured at normothermia suggest that ferroptosis can be triggered by lipid peroxidation not only in mitochondria but also at other sites within cells or at the plasma membrane at 37°C. First, two enzymes, NADPH-dependent flavin mononucleotide-containing cytochrome P450 oxidoreductase (POR) and NADH-cytochrome b5 reductase (CYB5R1), are necessary for ferroptosis in cancer cells that are susceptible to cell death (Zou et al., 2020; Yan et al., 2021). POR constitutes the cytochrome P450 (CYP) enzyme system and takes electrons from NADPH to donate it to CYPs, including cytochrome b5 or other redox partners, such as heme oxidase (HO1) and squalene monooxidase. However, the interactions between these proteins may not be essential for inducing ferroptosis. It has been proposed that POR and CYB5R1 directly donate electrons from NADPH/NADH to oxygen to generate H_2_O_2_, leading to lipid peroxidation of polyunsaturated fatty acid (PUFA) on the cellular membrane (Yan et al., 2021). Other organelles, such as peroxisomes or lysosomes, could also be the origin of ferroptosis triggers (Shintoku et al., 2017; Wenzel et al., 2017; Shah et al., 2018). Moreover, plasma membrane itself is a site of accumulation of lipid peroxides formed during ferroptosis, and the accumulated lipid peroxides facilitates cation permeability of the plasma membrane via the activation of the mechanosensitive non-selective cation channels, including piezo-type mechanosensitive ion channel component 1 (Piezo1) and the transient receptor potential (TRP) channel family, and inhibition of Na^+^/K^+^ -ATPase (Hirata et al., 2023). Clarifying the involvement of the plasma membranes and organelles other than mitochondria in cold-induced ferroptosis and cold resistance in hibernators awaits further investigation.

## Genetic basis of defenses against cold in hibernators

Accumulating evidence suggests that even at low temperatures, ROS can be generated within the cells of non-hibernators, leading to lipid peroxidation and cold-induced ferroptosis. Since mitochondria are thought to be the main source of ROS under cold conditions, it has long been studied whether mitochondria of mammalian hibernators exhibit different properties under cold stress in comparison with those of non-hibernators (Lyman et al., 1982; Brown et al., 2012; Mathers and Staples, 2019; Jensen et al., 2021). There are excellent reviews on the biochemical and metabolic properties of hibernators’ mitochondria and potential ROS management via sulfide metabolism (Giroud et al., 2020; Jensen and Fago, 2021). However, the exact mechanisms by which and to what extent hibernators suppress ROS generation and cold-induced ferroptosis still remains elusive.

One promising and powerful solution for clarifying the mechanisms of cold resistance is to directly manipulate genes. Comparative metabolomic analysis of hepatocyte-like cells derived from 13LGS-iPSCs revealed that 5-aminolevulinate (5-ALA), a metabolite produced in the first step of the heme synthesis pathway from glycine and succinyl-CoA, increased after 4 h of cold treatment, and is seemingly required for cold resistance of these cells because knockdown of 5-aminolevulinate synthase 1 (ALAS1) led to overproduction of ROS from mitochondria during cooling and rewarming (Zhang et al., 2022). Moreover, the authors demonstrated that liver organs harvested from rats treated at 4°C for 48 h with 5-ALA decreased cell stress and cell death markers in the process of rewarming compared to those without it. They suggested that 5-ALA enhances the activity of Complex III of mitochondrial, ETC, and thereby suppresses reverse electron transfer from Complex II to Complex I during the rewarming process. Thus, functional assessment of candidate genes that are identified by omics approaches could be a promising approach to elucidate mechanisms of cold resistance.

To identify and manipulate genes that can render cold resistance to hibernators and non-hibernators more directly, two pioneering papers conducted a gain-of-function screening using cDNA library generated from hibernators (Singhal et al., 2020; Sone et al., 2023). These gain-of-function strategies hypothesize that hibernators express genes that can render cold resistance even when heterologously expressed in non-hibernators that do not exhibit cold resistance. One study using a cDNA library from arctic ground squirrels (AGS) (*Spermophilus parryii*) tried to identify genes that confer resistance to mild cold (31°C) stress as well as ischemic stress and inhibition of the respiratory complex by the chemical inhibitor Rotenone in mouse neural precursor cells. An identified candidate was ATP5G1, which is a component of the ATP synthase complex in the mitochondria. AGS-ATP5G1 has multiple AGS-specific amino acid substitutions, and the human ATP5G1 protein, whose proline residue at position 32 is replaced with leucine mimicking the AGS gene (Hu ATP5G1P32L) confers resistance to mild cold stress (31°C) when overexpressed in mouse neural precursor cells. Replacement of the 32nd leucine of ATP5G1 in AGS neural precursor cells with a human-like amino acid (ATP5G1L32P) resulted in a slight decrease in stress resistance. Although the mechanism by which AGS ATP5G1 confers stress resistance is unknown, it is suggested to be related to an increase in the mitochondrial reserve respiratory capacity. It is unclear to what extent AGS-ATP5G1 contributes to the severe cold below 10°C that is experienced during hibernation, because the study used very mild cold stress (31°C) as a stressor. The 32nd leucine of ATP5G1 is not conserved in other hibernators, including Syrian hamsters (*Mesocricetus auratus*) (unpublished observation according to the uniport database), suggesting that it is the family sciuridae-specific mutation.

Another gain-of-function study identified genes that confer resistance to severe cold and rewarming experienced during hibernation (Sone et al., 2023). Through gain-of-function screening using a cDNA library prepared from Syrian hamster cancer cells that can survive even when exposed to severe cold (4°C) for more than 5 days and subsequent rewarming to 37°C, this study identified GPx4 as a strong suppressor of cold-induced ferroptosis in a non-hibernating human cancer cell line. As mentioned above, GPx4 is a protein that removes lipid hydroperoxides by reducing them with glutathione to lipid alcohol and plays a crucial role in suppressing ferroptosis. A cold-vulnerable human cancer cell line becomes resistant to severe cold (4°C) exposure and rewarming by heterologous expression of Syrian hamster GPx4, which suppresses lipid peroxidation. On the other hand, disruption of GPx4 in Syrian hamster cancer cells, a loss of function strategy, results in cold-induced ferroptosis after 3 days of cold exposure, judging from the hallmarks of ferroptosis, such as accumulation of lipid peroxides, necrotic morphology, and inhibition of cell death by iron chelators. Such a loss of cold resistance was rescued by overexpression of either hamster GPx4 or human GPx4. This study also demonstrates that GPx4 activity is necessary only under cold conditions because treatment with chemical inhibitors of GPx4 kills hamster cells only under cold treatment. These lines of evidence suggest that 1) GPx4 is an essential component of cold resistance in hibernator Syrian hamsters, and 2) both non-hibernator human GPx4 and hibernator Syrian hamster GPx4 are able to suppress cold-induced ferroptosis when overexpressed.

This study further identifies the factors that play a crucial role in cold resistance. Although GPx4-knock out (KO) hamster cells die after 5 days of cold conditions (4°C), they survive for 2 days at 4°C. This is in striking contrast to human cancer cells or mouse primary hepatocytes that die within 1 day under cold conditions. Such “short-term” cold resistance of GPx4-KO hamster cells is supported by other ferroptosis-suppressing pathways such as CoQ reducing system, biopterin synthesis system, and α-tocopherol (αT) (Sone et al., 2023). Single disruption of ferroptosis suppressor protein-1 (FSP1), dihydroorotate dehydrogenase (Dhodh), or GTP Cyclohydrolase 1 (Gch1), which are genes involved in CoQH_2_ or biopterin synthesis (Figure 1) (Bersuker et al., 2019; Doll et al., 2019; Kraft et al., 2020; Mao et al., 2021; Nakamura et al., 2023), does not cause cell death either at 37°C or at 4°C, but co-treatment with GPx4 inhibitors results in a massive increase in cell death at 4°C, particularly in the case of Gch1 disruption. These observations suggest that 1) ferroptosis-suppressing pathways act to inhibit cold-induced ferroptosis in an additive manner both in hibernators and non-hibernator cells, and that 2) among them, GPx4 is a central component of cold resistance in hibernators.

## Extrinsic and systemic factors that support cold resistance of hibernators

In addition to the above-mentioned cell-intrinsic mechanisms to prevent lipid peroxidation and cell death, hibernators also utilize extrinsic and systemic mechanisms supporting cold resistance to combat many types of oxidative stress *in vivo* accompanying hibernation. During both deep torpor and interbout arousal in 13LGS, the oxidation of lipids and proteins increases in various organs (Duffy and Staples, 2022). In AGS, the levels of antioxidant enzymes and low molecular weight ROS scavengers increase in the blood and cerebrospinal fluid during hibernation (Drew et al., 2002), and Vitamin C (ascorbate), a water-soluble antioxidant, increases 3- to 5-fold in the blood and 2-fold in the cerebrospinal fluid during deep torpor, whereas glutathione, another antioxidant, remains unchanged (Drew et al., 1999). More specifically, ascorbate levels decline during arousal from deep torpor (Toien et al., 2001). This occurs with the increase in urea, which is a degradation product of nucleic acids by xanthine oxidase, accompanied by the generation of free radicals and H_2_O_2_. This suggests that ascorbate is used to scavenge ROS in the blood and cerebrospinal fluid during arousal. Consistent with this hypothesis, a microdialysis analysis in hibernating Syrian hamsters demonstrated that the injection of an inhibitor of xanthine oxidase into the brain suppresses the consumption of ascorbate in the cerebrospinal fluid during the arousal period (Osborne and Hashimoto, 2007). Catalase activity to degrade H_2_O_2_ also increases in hamster blood during the mid-awake state and inhibits H_2_O_2_-induced cell death (Ohta et al., 2006). In contrast, it was reported that the levels of plasma ascorbate and urate are lowest during torpor and increase gradually during the arousal process in Syrian hamsters (Okamoto et al., 2006). The αT level peaked during deep torpor and declined during arousal. Because αT can be reduced from αT radicals by ascorbate, the opposite kinetics of ascorbate and αT in Syrian hamsters suggest that ascorbate may reduce αT radicals in the plasma during rewarming.

Ascorbate is synthesized mainly in the liver of most mammals, except primates, guinea pigs, and numerous bats (Duque et al., 2022). Ascorbate also regulates the iron homeostasis via intestinal iron absorption and promotion of cellular uptake of iron ion through Transferrin-TfR system (Lane and Richardson, 2014). Total iron levels in bones or livers in Daurian ground squirrels during DT is lower than those during summer (He et al., 2022; Yin et al., 2022). Transcriptomic analysis of white adipose tissues in Crossley’s dwarf lemurs and proteomic analysis of livers in 13LGSs demonstrated that expression levels of Ferritin is higher in torpor or the entry into torpor, respectively, when compared with those in active seasons (Rose et al., 2011; Faherty et al., 2018). Although the biological significance of these seasonal changes is unclear, such changes may help sequestering Fe^2+^ and contribute to inhibition of ferroptosis during torpor in hibernators.

**FIGURE 2:**
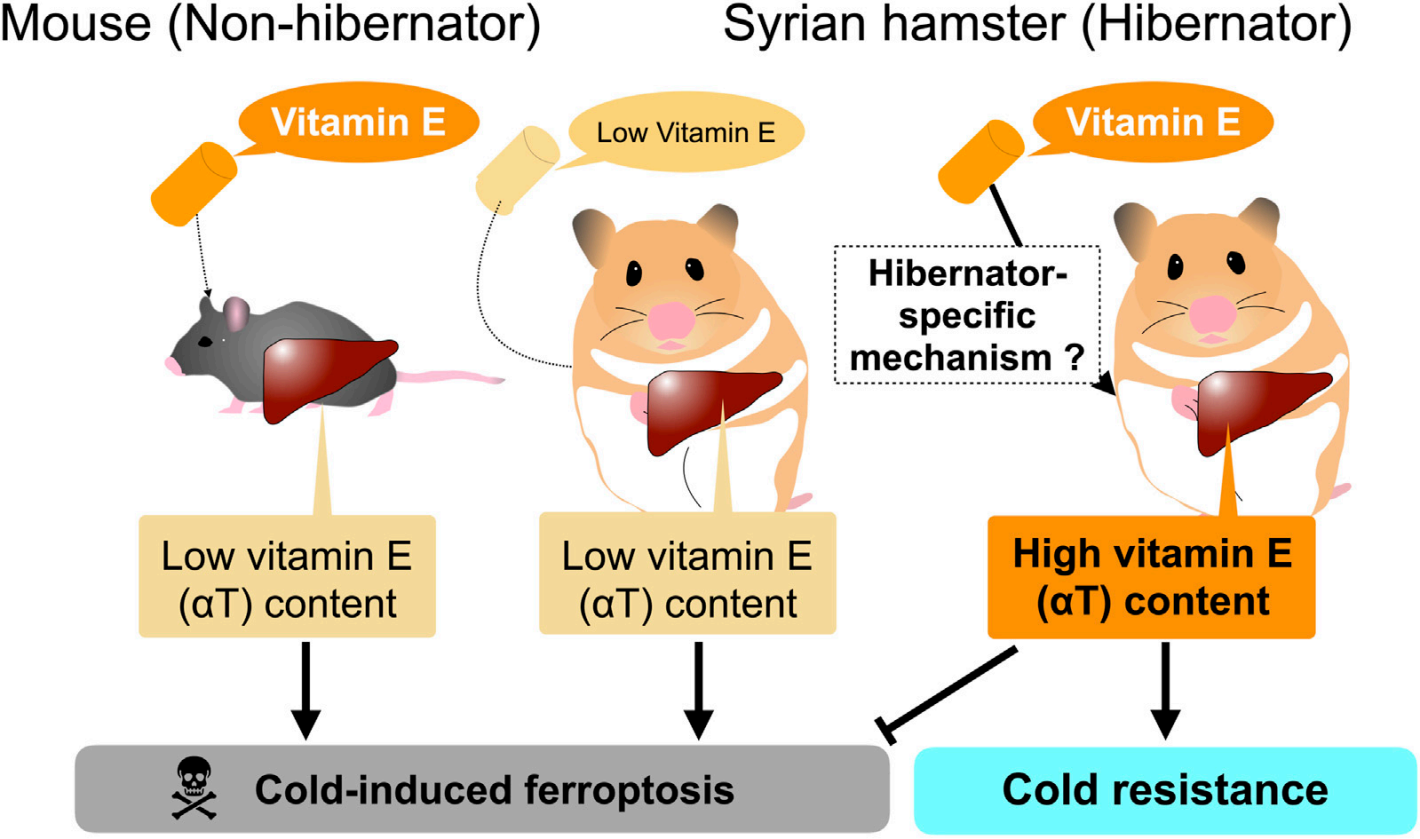
*In vitro* hepatic cold resistance affected by diet and species.

However, careful consideration is required to interpret the dynamics of systemic factors and their contribution to systemic cold resistance and redox status of animals. Most of the above-mentioned systemic factors are nutrients, metabolites, and minerals that are taken from diets and are synthesized, regenerated, and recycled within bodies, suggesting that their amounts could be affected by diets provided to the animals. For instance, the amount of αT in the liver and plasma of Syrian hamsters is largely affected by their diets given to them. αT is a major form of vitamin E obtained from the diet and plays a crucial role in rendering cold resistance to cells and organs by preventing lipid peroxidation. The liver is the primary organ responsible for storing and redistributing diet-derived αT from the portal vein into the systemic circulation. Redistribution of αT from the liver is achieved by αT transfer protein (α-TTP) in mammals, and loss-of-function mutations in humans result in low circulating vitamin E concentrations and many pathological symptoms related to vitamin E deficiency, such as neurodegenerative disorders (Di Donato et al., 2010; Kono et al., 2013). Primary cultured hepatocytes from Syrian hamsters survive for more than 5 days at 4°C, regardless of whether they are during the hibernation period or the non-hibernation period (Anegawa et al., 2021). Under the same conditions, primary cultured mouse hepatocytes die within 1–2 days at 4°C by cold-induced ferroptosis. The cold resistance of Syrian hamster hepatocytes depends on the diet of the animals used for and prior to hepatocyte culture (Figure 2). Diets containing relatively higher amounts of total vitamin E (more than >150 mg/kg; hereafter high αT diets) support hepatic cold resistance *in vitro*, while diets containing lower amounts of total vitamin E (less than <70 mg/kg; hereafter medium αT diets) fail to do so; Primary hepatocytes prepared from animals fed a medium vitamin E diet underwent cold-induced ferroptosis at 4°C. This loss of hepatic cold resistance was rescued by oral administration of αT. Hepatic αT content correlated well with cold resistance of hepatocytes. Interestingly, the high αT diets used in this study failed to increase hepatic αT content and render cold resistance in mouse hepatocytes, as mentioned above, indicating species differences in the hepatic ability to store dietary αT derived from diets. Thus, the cold resistance of primary cells prepared from tissues, at least in the liver, can be easily affected by species and diet fed to animals (Figure 2). Consideration of the possible effects of nutrients taken from diets on cold resistance may be useful in clinical applications, because αT could prevent lipid peroxidation that occurs not only in the deep torpor but also in the rewarming process during arousal, which is similar to the cold-rewarming process of cold-stored organs in the case of organ transplantation (Talaei et al., 2011; Vettel et al., 2014; Tolouee et al., 2022).

Contrary to the *in vitro* significance of αT in cold resistance, the *in vivo* significance of αT in hibernation is not yet clear. Vitamin E insufficiency causes many pathological conditions, hampering the examination of its role of vitamin E in hibernation. One preliminary study reported the negative effects of higher amounts of αT on torpor expression in golden-mantled ground squirrels (*Spermophilus lateralis*) using two diets that differ in tocopherol content (Frank et al., 2000), but it is difficult to conclude the role of tocopherols in torpor and hibernation because these diets are not chemically formulated and must differ in the content of many ingredients other than tocopherols, such as fatty acids and lipids. This is also true for the roles of other nutrients in torpor and hibernation in many literatures. For instance, it was hypothesized that omega-3 and omega-6 fatty acids play different roles in torpor and hibernation, based on studies in which phenotypes of torpor and hibernation are examined in animals fed diets containing different amounts of these fatty acids (Ruf and Arnold, 2008; Giroud et al., 2013; Arnold et al., 2015; Giroud et al., 2018; Giroud et al., 2020). However, such studies cannot be supported by fully chemically defined diets, and several reports contradict this hypothesis (Trefna et al., 2017; Rice et al., 2021). Obviously, as in the case of vitamin E, there must be many differences in its ingredients. Thus, future studies with strategies that directly manipulate the amount of these nutrients and redox factors within bodies, for instance, by genetic manipulation of genes responsible for the uptake, transport, or metabolism of specific nutrients and metabolites, are required to dissect the mechanisms of cold resistance at the whole-body level.

## Open questions and future perspectives

Owing to recent technological advances, it is now possible to address the mechanisms of cold resistance as well as other properties of hibernation from the functional assessment of genes in cells tissues, and hibernators (Singhal et al., 2020; Sone et al., 2023). It opens up new avenues for answering several fundamental and classic questions, as follows:

Q1: What genes are necessary and sufficient for cold resistance of hibernators?
Q2: Is it possible to confer cold resistance to non-hibernator cells?
Q3: Is cold resistance achieved either by a few specific genes or by the sum of modifications of the functions of many genes?
Q4: Are there common or diverse mechanisms for cold resistance among various hibernators?

Regarding Q1 and Q2, Sone et al. (2023) demonstrated that GPx4 is necessary for cell-intrinsic cold resistance in Syrian hamster cells and is sufficient to confer cold resistance heterologously to non-hibernator human and mouse cells when overexpressed. However, GPx4 is highly conserved among mammals, including non-hibernators, raising the question of why non-hibernators are vulnerable to cold, despite the presence of GPx4. Further investigation is needed to elucidate why non-hibernators are cold-vulnerable, and whether GPx4 function is specifically modified in hibernators. GPx4 is essential for survival of mice and humans. GPx4-deficient mice die during early development and mutations in GPx4 allele can be neonatally lethal (Imai et al., 2003; Yant et al., 2003; Cheff et al., 2021). GPx4 is also necessary for the survival of certain types of cancer cells (Yang et al., 2014), but GPx4-KO hamster pancreatic cancer cells can survive at 37°C, suggesting that other ferroptosis-suppressing pathways or redox pathways work to compensate for the loss of GPx4 in the cells at 37°C as well as under cold (Sone et al., 2023). Thus, hibernators may develop superior ability to non-hibernators in terms of redox regulation via ferroptosis-suppressing redox pathways such as GPx4 and its co-factors glutathione, CoQH_2_, BH4, and systemically supplied anti-oxidative factors such as αT or vitamin K (Mishima et al., 2022), thereby building up cold resistance. This idea may favor the later notion in Q3, when considering together with the report that diminishment of cold sensitivity in 13LGS and Syrian hamsters is due to species-specific amino acid substitutions of a cold-activated non-selective cation channel, TRPM8 (Matos-Cruz et al., 2017). Alternatively, the cellular membrane composition of hibernators may be intrinsically distinct from that of non-hibernators, or seasonally adapted to achieve cold resistance and maintain membrane fluidity under cold conditions, which could affect the structure of membrane proteins that are vital for maintaining cellular ion homeostasis (Aloia and Raison, 1989; Giroud et al., 2013; Cheff et al., 2021). Further efforts to answer these questions in detail will be necessary in specific model hibernators such as Syrian hamsters, in which the causal relationship between genes and phenomena can be functionally addressed, in combination with candidate genes and candidate molecules accumulated through the latest bioinformatics, omics, and comparative approaches in various hibernators, including ground squirrels, marmots, dormice, chipmunks, bats, and bears (Fedorov et al., 2014; Villanueva-Canas et al., 2014; Ferris and Gregg, 2019; Grabek et al., 2019; Jansen et al., 2019; Gillen et al., 2021; Chen and Mao, 2022; Bao et al., 2023; Christmas et al., 2023; Haugg et al., 2023; Heinis et al., 2023; Takamatsu et al., 2023; Thienel et al., 2023; Yang et al., 2023). Based on these studies, we will further clarify Q4 and its related question whether cold resistance associated with hibernation originates from a common ancestral trait or as a result of convergent evolution across distinct mammalian clades. In either case, understanding the mechanisms of cold resistance will expand our knowledge of thermal adaptation in mammals and merit possible medical applications to organ preservation and therapeutic hypothermia.

## Abbreviations

ROS, reactive oxygen species; ETC, electron transport complexes; αT, α-tocopherol; 13LGS, thirteen-lined ground squirrels; AGS, arctic ground squirrels.
